# Neuronal Survival, Morphology and Outgrowth of Spiral Ganglion Neurons Using a Defined Growth Factor Combination

**DOI:** 10.1371/journal.pone.0133680

**Published:** 2015-08-11

**Authors:** Jana Schwieger, Athanasia Warnecke, Thomas Lenarz, Karl-Heinz Esser, Verena Scheper

**Affiliations:** 1 Department of Otolaryngology, Hannover Medical School, Hannover, Germany; 2 Institute of Zoology, University of Veterinary Medicine Hannover, Foundation, Hannover, Germany; 3 Institute of Audioneurotechnology, Hannover Medical School, Hannover, Germany; 4 Cluster of Excellence Hearing4all, German Research Foundation, Hannover, Germany; Monash University, AUSTRALIA

## Abstract

**Objectives:**

The functionality of cochlear implants (CI) depends, among others, on the number and excitability of surviving spiral ganglion neurons (SGN). The spatial separation between the SGN, located in the bony axis of the inner ear, and the CI, which is inserted in the scala tympani, results in suboptimal performance of CI patients and may be decreased by attracting the SGN neurites towards the electrode contacts. Neurotrophic factors (NTFs) can support neuronal survival and neurite outgrowth.

**Methods:**

Since brain-derived neurotrophic factor (BDNF) is well known for its neuroprotective effect and ciliary neurotrophic factor (CNTF) increases neurite outgrowth, we evaluated if the combination of BDNF and CNTF leads to an enhanced neuronal survival with extended neurite outgrowth. Both NTFs were added in effective high concentrations (BDNF 50ng/ml, CNTF 100ng/ml), alone and in combination, to cultured dissociated SGN of neonatal rats for 48 hours.

**Results:**

The neuronal survival and neurite outgrowth were significantly higher in SGN treated with the combination of the two NTFs compared to treatment with each factor alone. Additionally, with respect to the morphology, the combination of BDNF and CNTF leads to a significantly higher number of bipolar neurons and a decreased number of neurons without neurites in culture.

**Conclusion:**

The combination of BDNF and CNTF shows a great potential to increase the neuronal survival and the number of bipolar neurons *in vitro* and to regenerate retracted nerve fibers.

## Introduction

Cochlear implants (CI) are the standard therapy for patients suffering from sensory neural hearing loss. In the last years, technical innovations like the development of novel speech processing strategies improved the benefits that patients can obtain with a CI, for example open speech recognition [1]. Nevertheless, there are still clinically relevant problems, which cannot be solved by technical advancements. Particularly around the electrode-nerve-interface, there are pathophysiological processes occurring, which can be manipulated via optimization of the biological aspects and combination of this approach to the technical device, the implant. The most important aspects, which hinder further improvement of CI outcome, are the retraction of the peripheral nerve fibers of the spiral ganglion neurons (SGN) following hair cell loss and the degeneration of the SGN in patients with long-time deafness.

Neuronal survival can be increased by electrical stimulation [2–7] or neurotrophic factors (NTFs). During embryonic development, two factors of the neurotrophin family, brain derived neurotrophic factor (BDNF) and neurotrophin-3 (NT-3), are involved in the synaptic connections between hair cells and SGN. Furthermore, BDNF, NT-3 and neurotrophin-4 (NT-4) increase neuronal survival of SGN. As previously summarized [8], the intracellular signaling pathways for BDNF are well examined. It acts by the activation of the tyrosine receptor kinase B (trkB) receptor (also named neurotrophic tyrosine kinase receptor type 2 (NTRK2)), which is present in different cell-types of the spiral ganglion [9]. Various downstream pathways promoting cell survival are activated when BDNF binds to the trkB receptors. One example is the activation of the phosphatidylinositol-3-kinase (PI3K), which finally induces the phosphorylation and thereby inactivation of pro-apoptotic targets [10]. A further important BDNF-trkB induced downstream pathway is the mitogen-activated protein kinase/extracellular-signal regulated kinase (MAPK/ERK) pathway resulting in the activation of cAMP-responsive element binding protein (CREB) [11]. Different co-factors in the divers signaling pathways that result in the activation of CREB are important determinants of the CREB-dependent gene targeting [12]. Based on the recruitment of such co-factors, the expression of specific genes may be induced by CREB [12]. A synergy between the neurotrophin family members and other NTFs may influence the recruitment of such co-factors.

For example, some of the neuropoietic cytokines (a cytokine subfamily of growth factors), including ciliary neurotrophic factor (CNTF) and leukemia inhibitory factor (LIF), have a positive effect on SGN survival and especially enhanced neurite outgrowth *in vitro* [13–18]. These cytokines are structurally unrelated to the neurotrophins and their effects are often additive or complementary [19]. Recent findings indicate an important role of CNTF in the postnatal hearing [20]. Bailey and colleagues demonstrated that it is expressed in the organ of Corti and the cochlear nucleus of rats with an increase concomitant with the hearing onset. They detected a higher expression of CNTF than BDNF. Also, CNTF and the CNTF specific receptor CNTF receptor α (CNTFRα) were found not only in the peripheral [20,21] but also in the central auditory structures [22]. Cell response to CNTF is mediated by a receptor complex consisting of the signal transducers glycoprotein130 and LIF receptor β (β-receptor components) and the CNTFRα ([23–26]; reviews: [26–30]). The dimerization of the β-receptor components results in a phosphorylation of Januskinases (JAK) [31] followed by signal transduction, including the signal transducer and activator of transcription (STAT) proteins [30,32]. The JAK/STAT pathway is considered to be the primary cytokine signaling pathway beside other pathways like the Ras-mitogen-activated protein (Ras-MAP) kinase pathway including ERK1 and ERK2 (MAPK/ERK kinase system) [33] and, cell line dependent, the phosphatidylinositol-3-kinase (PI3K) pathway (PI3K/Akt system) [30,34–36]. Alonzi et al. demonstrated that STAT3 and PI3K/Akt but not the MEK/MAPK signaling play a major role in mediating a survival response of neurons by cytokines [37] with STAT3, specifically activated by CNTF, leading to increased neuronal survival [38].

The activation of many downstream intracellular signaling molecules by theses neuropoietic cytokines overlap with the neurotrophin family members signaling pathways, but there are differences that could induce a synergistic interaction between these NTFs [26,27,39].

Resprouting of the peripheral processes of SGN would improve the performance of the CI, in particular by increasing the frequency specificity and decreasing the energy consumption of the implant. The need for neuronal attraction, guidance and regrowth towards an interfaceable device is important in all medical fields, from spinal cord injury and disorders of sensory systems therapy such as retinal and cochlear implants towards diseases of the central nervous system like Parkinson disease and neuroblastoma. Research in this field related to CI has just started and first studies indicate that it is possible to increase neurite growth pattern or even attract spiral ganglion dendrites out of the bony cochlear modiolus into the scala tympani where the cochlear implant is inserted. In all cases, neurotrophin family members such as BDNF [40,41] or BDNF in combination with NT-3 [42–45] or other NTFs like acidic fibroblast growth factor (FGF-1) [46] are used.

The neuroprotective and–regenerative effect of BDNF and CNTF has been described previously [14]. However, higher concentration of BDNF [15,47,48] and CNTF [35,47,49,50] were established for the best neuroprotective effects *in vitro* in the last years. Additionally, Bailey et al. 2014 [20] demonstrated that CNTF is expressed in a higher rate than BDNF in the inner ear.

In dissociated SGN cultures, neurons with different morphologies have been observed and it has been shown that NTFs influence their occurrence [16–18,50,51]. Instead of microexplants we therefore used a dissociated cell culture to analyze not only the survival rate but also the neuronal morphology and neurite outgrowth.

Thus, after verification of the specific receptors of the two NTF in our SGN culture, we adapted the experiments of Hartnick et al. to the current state of interest to detect a more detailed effect of the combination of BDNF and CNTF, which may be transferred to *in vivo* studies. Particularly, the influence on the survival, morphology and neuritogenesis of higher concentrations of these two NTFs (single and combined effect) in serum-free medium was investigated since maintenance and/or regeneration of a physiological bipolar morphology of the stimulated SGN is desirable in CI related research.

## Materials and Methods

### Ethics statement

The experiments and analysis of this study were conducted from spring to autumn 2014. They were performed in accordance with the institutional guidelines for animal welfare of the Hannover Medical School following the standards described by the German ‘Law on Protecting Animals’ (Tierschutzgesetz) and with the European Directive 2010/63/EU for protection of animals used for experimental purposes. The euthanasia for our *in vitro* experiments is registered (no.: 2013/44) with the local authorities (Zentrales Tierlaboratorium, Laboratory Animal Science, Hannover Medical School, including an institutional animal care and use committee) and reported on a regular basis as demanded by law. For exclusive sacrifice of animals for tissue analysis in research, no further approval is needed if no other treatment is applied beforehand (§4). The rats were bred and born for research study purposes. A breeding stock was supplied by Charles River (Charles River, Wilmington, USA) and housed with their litters in the facilities of the licensed Institution of Laboratory Animal Science of the Hannover Medical School. To minimize the stress level for the neonatal rats, they were euthanized by decapitation prior to any experimentation by a licensed person.

### Spiral ganglion cell preparation

Postnatal Sprague Dawley rats (P2-4) of both sexes were dissected for tissue harvesting for cell culture experiments. After decapitation, skin and skull were opened along the midline and the brain removed after extension of the center cut. Back half of the skull including the temporal bones was ventrally cut in midline, removed and transferred into a Petri dish (60 x 15 mm; BD Falcon, Sparks, USA) with ice-cold Ca^2+^/Mg^2+^-free D-phosphate-buffered saline (PBS) (Invitrogen, Karlsruhe, Germany). The following preparation of the spiral ganglion was performed under microscopic control (Leica KL 1500 LCD; Leica, Wetzlar, Germany). First, the temporal bone was identified and the bony cochlear capsule carefully removed to provide a good visibility of the membranous labyrinth of the cochlea. This membranous cochlea was removed and transferred to another Petri dish with ice-cold PBS. Under higher magnification the membranous capsule was removed and the stria vascularis and the organ of Corti connected to the spiral ganglion were detached from the modiolus. Finally, the entire spiral ganglion was separated from the two other strands and collected in ice-cold Ca^2+^/Mg^2+^-free Hank’s balanced salt solution (HBSS) (Gibco Invitrogen, Karlsruhe, Germany). All preparations were performed with forceps (Dumont #3, #5; Fine Science Tools, Heidelberg, Germany).

The enzymatical and mechanical dissociation of the spiral ganglion was performed as previously described [48]. HBSS mixed with 0.1% trypsin (Biochrom, Berlin, Germany) and 0.01% DNase I (Roche, Mannheim, Germany) was added to the spiral ganglion (30–40 ganglia/2 ml) and incubated at 37°C for 16 minutes (with intermittent shacking). Afterwards the cells were centrifuged by short spin, the supernatant discarded and the enzymatic dissociation stopped by addition of 200 μl fetal calf serum (FCS) (Invitrogen, Karlsruhe, Germany). This was followed by threefold addition and removal of 1 ml of serum-free culture medium (see below) to wash out FCS, trypsin and DNase. Now, the digested cells were filled up to 1 ml with culture medium (see below) and mechanically dissociated first with a 1000 μl pipette-tip followed by a 200 μl pipette-tip (TipOne; StarLab, Hamburg, Germany). The ganglia were triturated until no cell clusters were visible in the suspension. After determining the cell number in a Neubauer chamber (BRAND GmbH, Wertheim, Germany) using the trypan blue (Sigma Aldrich, Taufkirchen, Germany) exclusion test, the cell suspension was diluted with serum-free culture medium (see below) to a concentration of 1.4 x 10^4^ cells/50 μl.

### Spiral ganglion neuron cultivation

The spiral ganglion consists of an inhomogeneous cell population including beside neurons non-neuronal cells like glial cells (e.g. Schwann cells) and fibroblasts. Thus, the term spiral ganglion cells (SGC) refers to all cell types. After neuron-specific staining, spiral ganglion neurons (SGN) were identified and quantified and were included for the morphometric analysis. Cultivation of the dissociated SGC was performed in 96-multiwell culture plates (Nunc A/S, Roskilde, Denmark), previously coated with poly-DL-ornithine (0.1 mg/ml; Sigma Aldrich, Taufkirchen, Germany) and Laminin (0.01 mg/ml; Naturel Mouse Laminin; Invitrogen, Karlsruhe, Germany).

The serum-free culture medium consisted of Panserin 401 (PAN BIOTECH, Passau, Germany) supplemented with 1M Hepes-buffer solution (Invitrogen, Karlsruhe, Germany), PBS, penicillin (30 U/ml; Biochrom, Berlin, Germany), glucose (40%/ml; B.Braun, Melsungen, Germany), insulin (4 mg/ml; Biochrom, Berlin, Germany) and N2-supplement (Invitrogen, Karlsruhe, Germany).

Cells were seeded in a concentration of 1.4 x 10^4^ cells/well. The negative control (NC) contained no NTFs, BDNF (recombinant human BDNF, Invitrogen, Karlsruhe, Germany) was added to the SGC culture in a concentration of 50 ng/ml, CNTF (recombinant human CNTF, Novoprotein, Summit, USA) in a concentration of 100 ng/ml and both factors in combination with the same concentrations. The cells were cultivated in an incubator (CB 150 E3; Binder, Tübingen, Germany) at 37°C, 5% CO_2_ and 95% humidity for 48 h. The seeding control was fixed after 4 h of incubation in serum free culture medium. Fixation of all cells was performed with a 1:1 acetone (J. T. Baker, Deventer, Netherlands)/methanol (Roth, Karlsruhe, Germany) solution for 10 minutes.

Cell culture for receptor-immunocytochemistry was performed in the same way, but the cell culture medium contained 10% FCS and cells were fixed with 4% paraformaldehyde (PFA, Merck Millipore, Darmstadt, Germany; mixed in PBS) for 10 minutes at room temperature.

For storage in PBS or prior to staining, cells were washed three times with PBS.

### Immunocytochemistry

Since we were interested in the receptor expression profiles for the NTFs under the present culture conditions, we performed immunocytochemistry of the CNTF receptor CNTFRα and the high affinity receptor for BDNF, the trkB-receptor. All antibodies used are listed in Tables 1 and 2.

**Table 1:** Primary antibodies.

Antibody	Antigen	Host, type, dilution	Company, cat.-no
Mouse monoclonal antibody Neurofilament 200 kD	200 kD Neurofilament	Mouse, monoclonal, 1:500	Novocastra, #NCL-NF200
Rabbit polyclonal anti-CNTF receptor alpha	Ciliary Neurotrophic Factor Receptor (N-Term)	Rabbit, polyclonal, 1:300	Bioss, Bs-1516R
Rabbit polyclonal anti-trkB	trkB	Rabbit, polyclonal, 1:50	Abcam, ab18987
Chicken polyclonal anti-200 kD neurofilament heavy antibody	200 kD Neurofilament Heavy	Chicken, polyclonal, 1:1000	Abcam, ab4680

Primary antibodies used for the staining of spiral ganglion neurons.

**Table 2:** Secondary antibodies.

Antibody	Antigen	Host, type, dilution	Company, cat.-no
Biotinylated horse anti-mouse IgG	Mouse IgG–H&L	Horse, 1:2000	Vectastain Elite ABC-kit, Vector Laboratories #PK6100
Goat anti rabbit IgG (H+L), Alexa Fluor 594	Rabbit IgG–H&L	Goat, polyclonal, 1:200	Jackson ImmunoResearch, #111-585-144
Goat anti-chicken IgY (H+L), DyLight 488	Chicken IgY–H&L	Goat, polyclonal, 1:200	Abcam, ab96947

Secondary antibodies used for the staining of spiral ganglion neurons.

The cells were stained with polyclonal chicken anti 200-kD neurofilament to distinguish neurons from the non-neuronal cells. Cells were first permeabilized with PBS and 0.5% Triton X-100 (Sigma Aldrich, Taufkirchen, Germany) for three minutes at room temperature. After rinsing with PBS containing 0.1% Triton X-100 (PBT) two times, cells were blocked for one hour with PBT and 5% FCS. After thrice washing with PBT, cells were incubated with the primary antibodies (anti-trkB, anti-CNTFRα and anti-200-kD neurofilament) diluted in antibody dilution buffer (2% FCS and 1% BSA in PBS) over night at 4°C. Next day, the primary antibodies were washed out three times for five minutes with PBT and secondary antibodies in antibody dilution buffer were added for one hour. Finally, the secondary antibodies were rinsed with PBT again and the stained cells were stored in darkness. Standardly, negative controls were performed by omitting primary antibodies. Images were taken with an inverse microscope (Axio; Carl Zeiss, Jena, Germany) using an Axio Cam MRm (Carl Zeiss, Jena, Germany) with Axio Vision-software (Carl Zeiss, Jena, Germany).

For analysis of survival rate, neuronal morphology and neurite length, neurons were stained against the 200-kD neurofilament. The protocol was described in detail previously [48]. In summary, a monoclonal mouse 200-kD neurofilament primary antibody was used followed by Vectastain Elite ABC-kit (Vector Laboratories, Burlingame, USA) and peroxidase DAB substrate (Peroxidase Substrate Kit DAB; Vector Laboratories, Burlingame, USA) for visualization.

### Cell counting, neuronal morphology and neurite length measurement

Cell counts and images of the cell culture were performed using an inverted microscope (Olympus CKX 41, Hamburg, Germany).

Survival rate, neuronal morphology and neurite length were analyzed in three independent experiments. Each condition was performed in triplicate per experiment and was analyzed for survival rate and neurite length. One of the three wells was representatively used to classify the different neuronal morphologies of the SGN.

Surviving SGN were defined as cells stained positive for neurofilament and with neurites at least three times longer than the soma diameter [47]. For all experimental groups, the number of surviving neurons per well was counted and given as percentage of the average number of neurons in the seeding control.

For neuronal morphology, the SGN were examined and counted. According to Whitlon et al. 2007 they were classified into following groups: monopolar, bipolar, multipolar, pseudomonopolar and no neurites [50]. Cells that were not clearly identified as well as cells in clumps were not counted. For analysis, the percentages of different morphologies were calculated from the total number of counted neurons. For detailed description see Whitlon et al. 2007 [50].

For the analysis of the SGN neurite length, pictures were taken with a CCD-camera (Colorview XS, SIS, Münster, Germany) from at least five fields of view per well [47] and processed using an image analysis program (CellP, SIS; Olympus). The 15 longest neurites of these five fields of view (3 neurites/ field) were measured using the polygon function of the image analysis program followed by statistical analysis. For wells with less than 15 neurons in total, the longest neurite of every neuron was measured. If one cell presented several neurites, the longest neurite was analyzed [47,52,53].

### Statistical analysis

The data (means ± SEM) were analyzed for statistical significance using one-way analysis of variance (ANOVA) followed by Newman-Keuls Multiple Comparison Test using GraphPad Prism. *P* values less than 0.05 were considered statistically significant.

## Results

The SGC culture is a mixture of SGN and surrounding non-neuronal cells. In this study we mainly focus on the SGN.

### Immunocytochemistry of growth factor receptors

**Fig 1:**
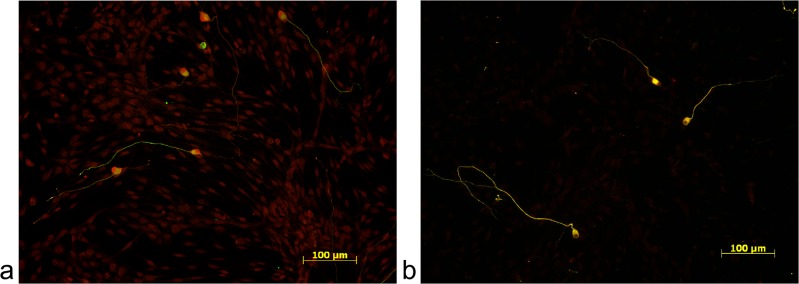
Receptor detection. Both, the trkB receptor (a) and CNTFα receptor (b) were detected in the cultures of dissociated spiral ganglia. To verify the presence of the receptors (labeled in red) on SGN, the neuronal cells were double stained with neuron-specific 200 kD anti-neurofilament antibody (labeled in green).

By immunocytochemistry, the CNTFRα and the trkB receptor were detected in the SGC culture. Both receptors were expressed on the cell body and neurites of SGN as well as on the surrounding non-neuronal cells (Fig 1).

### Survival rate

**Fig 2:**
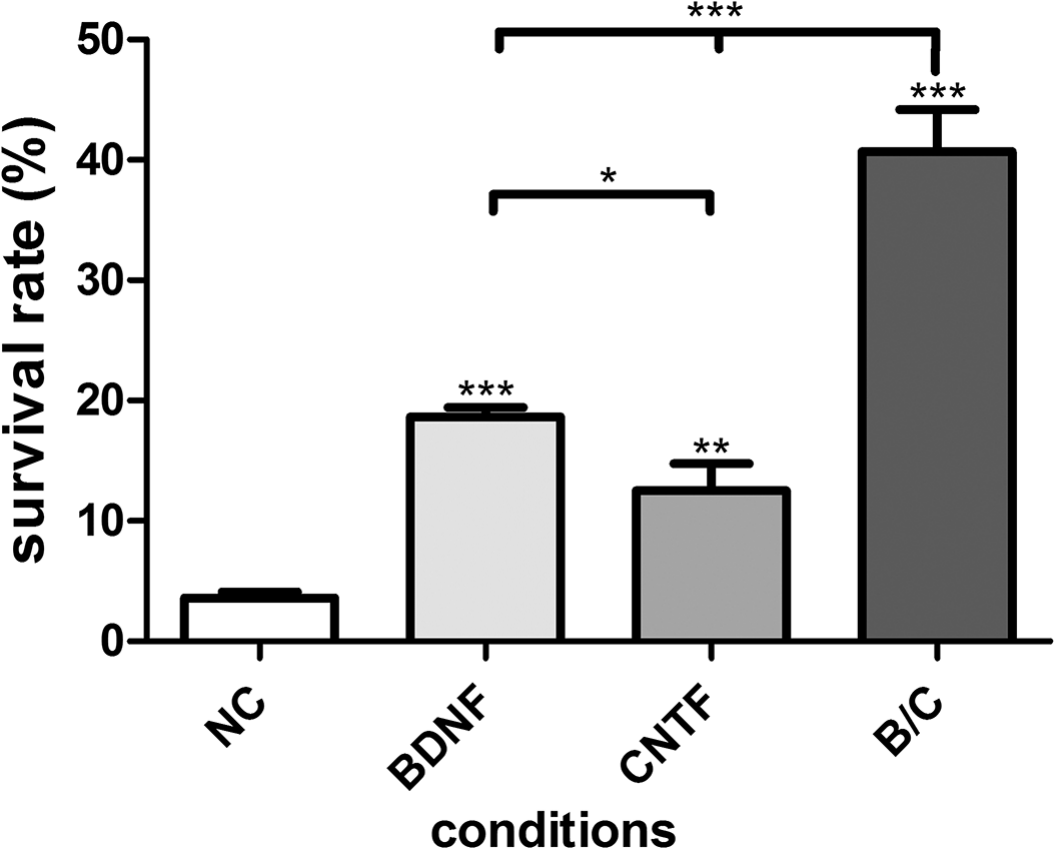
Neuronal survival Effect of 50 ng/ml BDNF, 100 ng/ml CNTF and the combination of both factors (B/C) compared to the negative control (NC) on spiral ganglion neurons (SGN) survival rate after 48 h *in vitro* incubation. The survival rate is given as a percentage of the initially seeded cells per experiment which was set as 100%, represented as mean and standard error of mean (SEM). Asterisks above the error bars indicate the significance of the conditions compared to the negative control. Both neurotrophic factors improved the survival rate significantly and the combination of BDNF and CNTF resulted in the significantly highest increase of surviving neurons (*p<0.05; **p<0.01; ***p<0.001; independent experiments: 3, wells per experiment: 3).

The number of seeded neurons (seeding density) was determined after 4 hours of incubation. After neuron-specific staining of the seeding control, 805.6 ± 90 neurons/well were amounted. The survival rate in each condition was calculated by relating the number of survived neurons to the mean seeding density of the respective plate. After 48 hours of incubation, the untreated (negative) control showed a survival rate of 3.57 ± 0.52%. Compared to the negative control, the neuronal survival in wells treated with 50 ng/ml BDNF (18.64 ± 0.79%) or 100 ng/ml CNTF (12.51 ± 2.27%) was significantly increased (BDNF: *p*<0.001, CNTF *p*<0.01). The neuroprotective effect of BDNF was significantly higher than that of CNTF (*p*<0.05). The significantly highest neuronal survival rate was observed in SGN treated with the combination of 50 ng/ml BDNF and 100 ng/ml CNTF with 40.69 ± 3.52% (*p*<0.001) compared to all other conditions (Fig 2).

### Neuronal morphology

**Fig 3:**
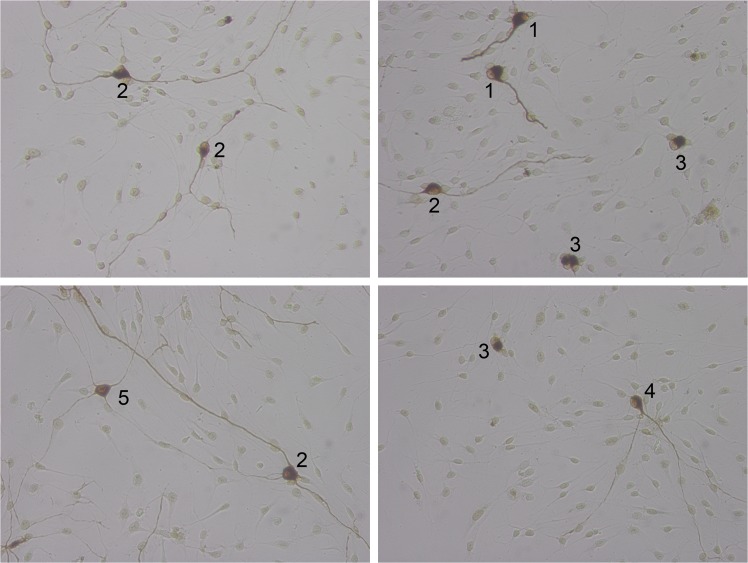
Classification of neuronal morphologies Different morphologies of SGN could be detected in the culture. 1: monopolar neurons; 2: bipolar neurons; 3: neurons with no neurites, 4: pseudomonopolar neurons; 5: multipolar neurons. Neurons were stained with DAB against neurofilament. Magnification: 200x.

**Fig 4:**
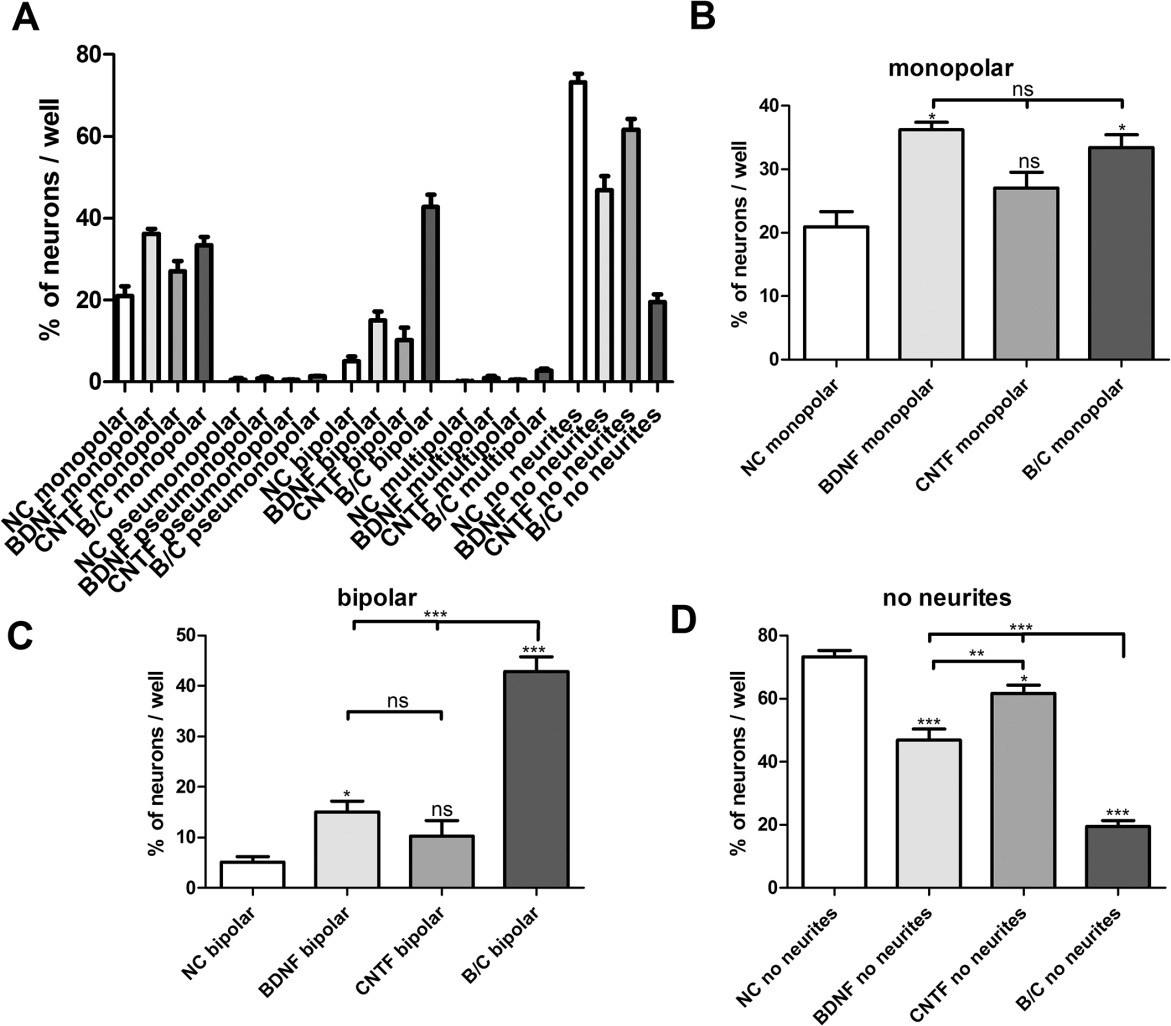
NTF effect on neuronal morphologies Effect of 50 ng/ml BDNF, 100 ng/ml CNTF and the combination of both NTFs (B/C) compared to negative control (NC) on neuronal morphology in terms of polarity and missing neurite outgrowth after 48 h *in vitro* incubation. All morphologies and their distribution dependent on the treatment are depicted in an overview graph (mean ± SEM) (A). The analysis of the most frequent neuron types is depicted in B-D. The amount of monopolar neurons was increased when treated with BDNF or both NTFs. Only BDNF (15.04%) but not CNTF (10.21%) increased the amount of bipolar neurons. Both factors added simultaneously resulted in a highly significant increase to 42.84% of bipolar neurons compared to the negative control, showing a synergistic effect. The number of neurons without neurites was significantly decreased when both NTFs were administered together. BDNF or CNTF treatment resulted in a statistically lower percentage of neurons without neurites compared to the negative control as well. Furthermore, BDNF treatment caused a lower rate of neurites without neurons than CNTF. The SGN morphology is given as a percentage of the total number of scored neurons represented as mean and standard error of mean (SEM). Asterisks above the error bars show the significance of the conditions compared to the relevant negative control. (mean ± SEM; *p<0.05; **p<0.01; ***p < 0.001; ns = not significant; independent experiments: 3, wells per experiment: 1).

Neurons were classified as bipolar, monopolar, multipolar, pseudomonopolar or with no neurites and were related to the total number of the scored neurons. Fig 3 depicts representative neurons for each classification. Analyzing the morphology of the SGN (see Figs 3 and 4) in relation to the treatment with NTFs, some differences were observed: the pseudomonopolar and the multipolar neurons represent only a small percentage of the neuronal population (all below 5%) and there is only one significant difference between the condition groups in multipolar morphology (combination of BDNF and CNTF caused significant more multipolar neurons compared to the other conditions, p< 0.01; significance not marked in Fig 4). In our experiments, the monopolar neurons represented a high proportion of all neurons. Compared to the negative control (20.93 ± 2.42%), there was a significantly higher rate of monopolar neurons after treatment with BDNF (36.23 ± 13.2%, *p*<0.05) and the combined NTFs (33.43 ± 2.02%, *p*<0.05) but not with CNTF alone (27.06 ± 2.50%). Between the NTF-treatment groups, there was no significant difference. By contrast, the number of bipolar neurons is highly significantly increased (*p*<0.001) when BDNF and CNTF were added in combination to the culture (42.84 ± 2.94%) compared to the negative control (5.12 ± 1.09%) and to treatment with each factor alone (BDNF: 15.04 ± 2.14%; CNTF: 10.21 ± 3.09%). Compared to the negative control, the number of bipolar neurons was significantly increased only after treatment with BDNF (*p*<0.05), but not after treatment with CNTF. In cultures treated with both growth factors, a significantly decreased number of neurons with no neurites (19.50 ± 1.85%) was determined in comparison to all other groups (NC: 73.24 ± 2.04%, BDNF: 46.86± 3.54%, CNTF: 61.63 ± 2.68%; *p*<0.001). Compared to the control, the number of neurons with no neurites was significantly lower in cultures treated either with BDNF (*p*<0.001) or CNTF (*p*<0.05).

### Neuronal outgrowth

**Fig 5:**
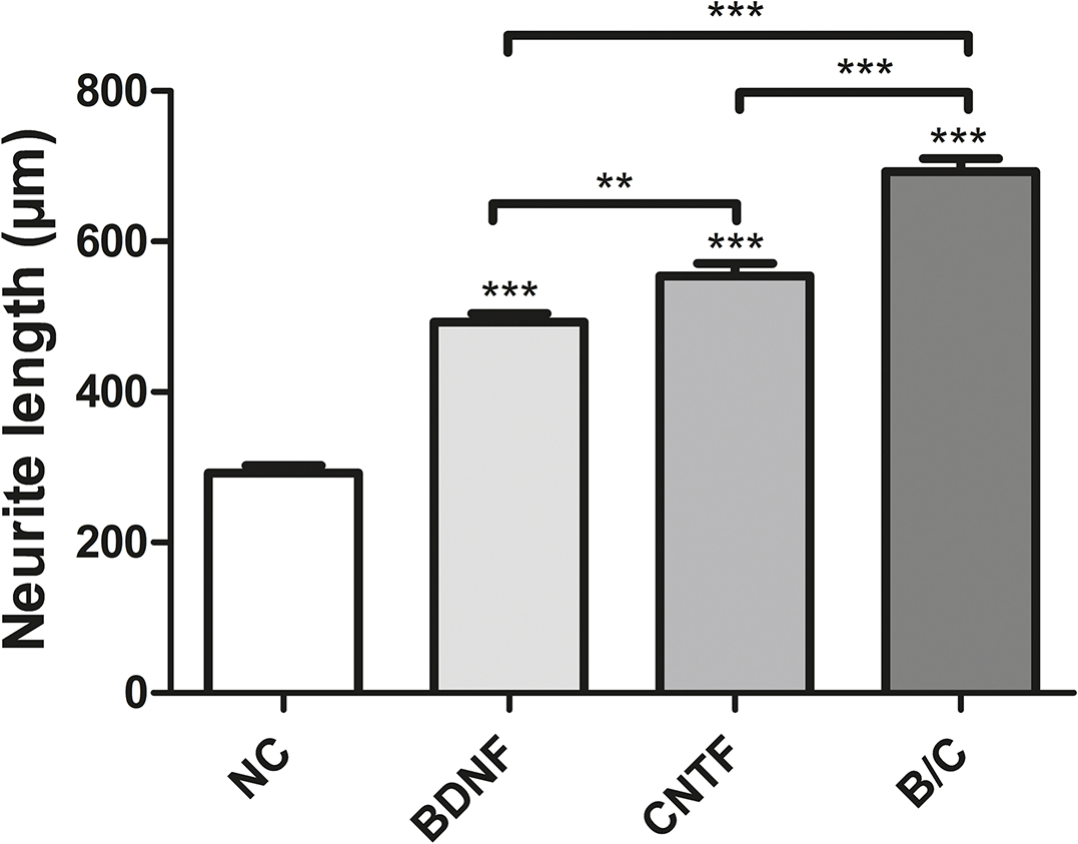
Neurite outgrowth Neurite length (μm) was measured on spiral ganglion neurons (SGN) cultured for 48 h with addition of growth factors (50 ng/ml BDNF, 100 ng/ml CNTF and combination of both (B/C)) or without factor application (negative control, NC). All neurotrophic factor treatments caused significantly longer neurites compared to the neurons of the negative control. The significantly longest neurites were detected when BDNF and CDNF were applied concurrently. Asterisks above the error bars indicate the significance in the experimental conditions compared to negative control (**p<0.01; ***p < 0.001; independent experiments: 3, wells per experiment: 3).

Neurite length was measured as an indicator of neuronal outgrowth and given as mean ± SEM for all four conditions (Fig 5). All conditions containing NTFs led to significantly (*p*<0.001) longer neurite outgrowth when compared to the negative control (292.4 ± 10.24 μm). The SGN-medium enriched with CNTF caused a neurite outgrowth of 554.4 ± 16.52 μm and thus significantly longer neurites then BDNF enriched medium (493.0 ± 11.44 μm; *p*<0.01). The highest neuritogenesis was detected after applying the combination of BDNF and CNTF resulting in a length of 692.8 ± 17.70 μm, which is a significantly increased neuronal outgrowth when compared to the other experimental conditions (*p*<0.001). Neurite length is listed and summarized as mean, minimum and maximum for the different conditions in Table 3.

**Table 3:** Neurite length.

	NC	BDNF	CNTF	BDNF + CNTF
Mean	292.4	493	554.4	692.8
Minimum	99.27	247.2	107.2	345
Maximum	657.7	941.5	1163	1523

Neurite length presented as mean, minimum and maximum, given in μm (independent experiments: 3, wells per experiment: 3).

## Discussion

Our data demonstrate that a combined treatment with BDNF and CNTF in higher concentrations has a beneficial effect on neuronal survival and neurite outgrowth and affects the number of bipolar neurons and neurons with no neurites on rat SGN *in vitro*.

By immunocytochemistry, the receptors for BDNF and CNTF were detected in dissociated SGC. There might be a difference in the expression of the two receptors. The CNTFRα seemed to be mainly expressed on the SGN. Only a part of the surrounding non-neuronal cells was labeled positive for the CNTFRα with a lower intensity. By contrast, the trkB receptor was detected on neurons and surrounding non-neuronal cells. Here, the positively labeled cells appeared to have a more intense fluorescence signal compared to CNTRα immunolabeling. Since the CNTFRα is expressed mainly neuronspecific, this finding is in accordance with the literature [27] and implies a possible difference in target cells and reaction between the two factors.

In 1996, Hartnick et al. [14] tested BDNF, CNTF and their combination on dissociated acoustic neurons of neonatal rats with best results for survival at concentrations of 10 ng/ml for BDNF and 0.01 ng/ml for CNTF. In this study, both factors promoted the survival of dissociated SGN and the neurite outgrowth of acoustic ganglion microexplants. BDNF had the strongest effect on neuronal survival, while CNTF was more potent in stimulating the neuritogenesis. This is corroborated by our finding. In combination, BDNF and CNTF promoted neuroprotection and neuritogenesis in an additive manner. Interestingly, CNTF exerted its effect only when applied at low concentrations (Hartnick et al.). However, in our experimental setting we observed an increased survival rate after application of high doses of BDNF and CNTF. This amounted to eleven times higher survival rate when compared to the negative control and was in contrast to Hartnick et al., who detected only a four-fold increase of the survival rate. An enhancement of factor concentrations may thus result in a better neuroprotection.

The neurite length as indicator for neuritogenesis was analyzed in dissociated SGN. Therefore, the results are difficult to compare with the findings on microexplants of the acoustic ganglion and the measured ratio of neurite length to explant diameter used by Hartnick et al. In our study, the combination of both factors was highly beneficial on the neuritogenesis compared to all other tested conditions, which is consistent with Hartnick’s findings. While Hartnick et al. detected an additive effect of the combined NTFs on neurite outgrowth, we observed a synergistic effect with twice longer neurites when adding both NTFs compared to the negative control. Analyzing 15 neurites of each well may account for these differences when compared to the inclusion of all regenerated neurites in the analysis as performed in the experiments of Hartnick et al. using microexplants. In addition, the different basal media (DMEM vs Panserin) used might also have an effect on neuritogenesis [54]. Another possibility could be the higher doses of BDNF. Jin et al. [18] detected an inhibitory effect on neurite outgrowth, when adding 50 ng/ml BDNF, which disappeared by reducing the BDNF to 10 ng/ml. Howbeit we demonstrated a significant increase of neurite outgrowth even by using 50 ng/ml BDNF.

An additional finding in the present study was the influence of BDNF and CNTF on the morphology of the neurons in the SGC culture. The normal morphology of the SGN type I, connecting the inner hair cells (dendrite) with the brain stem (axon), is bipolar [9,51,55]. In our experiments, we demonstrated that the combination of BDNF and CNTF leads to a significantly higher number of bipolar neurons in culture. With the combined NTFs, the percentage of bipolar neurons was more than eight times higher than in the negative control and both factors worked in a not only additive but in a highly synergistic manner. By contrast, we detected a decrease of the neurons without neurites by adding the NTFs. Here, the combination of BDNF and CNTF worked in a synergistic manner again. The number of monopolar neurons is slightly increased by the addition of BDNF and the combined NTFs.

Whitlon et al. detected similar results for the neuropoietic cytokines LIF [51], CNTF and hOSM (human recombinant oncostatin M) [50]. In their experiments from 2006, they measured that 90% of P1 mouse SGN were of a monopolar or bipolar morphology, when plated with NT-3 (8 ng/ml), BDNF (8 ng/ml), LIF (80–100 ng/ml) and serum (10%) and only very few had no neurites, or a multipolar or pseudomonopolar morphology. LIF increased the number of surviving neurons and this increase was due to a raise of the number of bipolar neurons, which means that LIF preferentially affects the bipolar morphology of SGN. They suggest that LIF rescues an additional number of neurons in culture, which had a bipolar morphology in the presence of the NTFs and serum. In 2007, they confirmed these effects on the morphology for CNTF and hOSM. CNTF (50–100 ng/ml) and hOSM (40–100 ng/ml) (+ BDNF, NT-3 and serum) increased the survival of SGN associated with a significant increase in the absolute number of bipolar neurons (CNTF: about 16%), but no significant effects on the number of monopolar neurons (CNTF: about 17%) and those without neurites (CNTF: about 3%). They hypothesize that the cytokines do not induce a regrowth of the second process of a monopolar neuron, since the absolute number of monopolar neurons does not change with or without LIF.

Compared to Whitlon et al. 2007, we detected a significant reduction in the number of neurons with no neurites after application of BDNF and CNTF. This effect was even higher, when CNTF was combined with BDNF. This implicates that the regeneration of two neurites is induced by the combination of BDNF and CNTF resulting in increased numbers of bipolar neurons and reduced numbers of neurons without neurites. Like Whitlon et al., we detected only a small effect on the number of monopolar neurons in the different conditions. Even in the NC the number of monopolar neurons is still high compared to the number of bipolar neurons. This might indicate that the monopolar neuronal morphology is less sensitive to serum and NTF withdrawal. And it might support the hypothesis that there are different populations of neurons in the SGC culture. Some can only regenerate one neurite, showing a monopolar morphology in culture. Others are able to regenerate two neurites and become bipolar, when treated with BDNF and CNTF. The culture includes type I and type II SGN so there are at least some differences in the neurons.

The results for the combined NTFs in our study differ from those of Whitlon et al. 2007 [50]: 40% vs 90–100% survival rate at Whitlon, 43% vs 16% bipolar neurons and 20–70% vs under 5% (for all conditions) neurons without neurites. One difference between the two studies is the species used. Different values for type I and type II SGN were given for neonatal rats and mice [56]. Another influence accounting especially for the differences in survival rate and neurons with no neurites could be the use of serum-deprived medium in our study. By contrast, Whitlon et al. used basal medium enriched with serum for their investigation. However, a distinct increase of surviving neurons has been described after the combined use of BDNF and serum when opposed to serum-free medium complemented with BDNF [54]. Moreover, Whitlon et al. used a cocktail of factors by combining the application of BDNF and CNTF with NT-3. The use of growth factor cocktails very potently increases the survival rates of neonatal rat SGN even without addition of serum as has been demonstrated recently [53]. However, our experiments focused on the single as well as the combined effect of BDNF and CNTF rather than the use of factor cocktails.

Like Whitlon et al. 2007 already stated, the use of dissociated cultures and the investigation of the different neuronal morphologies allows the examination of biochemical and other interventions that can shift a predominantly monopolar population to a bipolar population of SGN, a conversion that may have implications in the whole animal [50]. Thus, our findings for a defined NTF application in a serum-free medium may offer an advantage for *in vitro* models.

A combination of BDNF and CNTF may specifically induce the maintenance of the physiological bipolar morphology and regeneration of the nerve fibers. Since the CI stimulates the type I SGN, it is of major importance, to regenerate the retracted dendrites of the SGN [40–42,44]. Whitlon et al. 2007 proposed that the situation *in vivo* in a damaged cochlea with degenerated hair cells and retracted peripheral neurites of the SGN (in particular, if the central axon remains connected to the brain stem, as cochlear implant function would indicate) could potentially arrive at a state similar to the monopolar neurons in culture [50]. Liu et al. [57] verified this assumption in 2015. By SEM (scanning electron microscopy) and 3 D reconstruction of a human spiral ganglion of a patient with noise-induced hearing loss, they histologically verified that more than half of the investigated type I SGN in the Rosenthal`s canal were monopolar. They also suspected that a drug-induced re-sprouting of the dendrites could improve the functional results of a CI.

Shinohara et al. [58] (immediate NTF-treatment after deafening) and Yamagata et al. [59] (2 to 6 weeks delayed NTF-treatment after deafening) tested the combination of BDNF and CNTF *in vivo*. After deafening, guinea pigs were implanted with an electrode and (direct or delayed) a mini osmotic pump system infusing BDNF (100 μg/ml) and CNTF (100 ng/ml) to the inner ear for one month. Both SGN survival and eABR (electrically evoked auditory brain stem responses) were evaluated. When treated with the NTFs immediately after deafening, the number of surviving SGN was significantly increased and significant differences in eABR thresholds in the control group with artificial perilymph and BDNF plus CNTF were observed. Also, after delayed treatment with a greater degeneration of the SGN, the NTFs enhanced the survival of the auditory neurons. In both experiments, the NTF application rescued neurons from death and enhanced the electrical excitability even with a reduced cell population after 6 weeks of degeneration. They suggested that the improved threshold sensitivity may reflect the regrowth of SGN peripheral processes due to the NTF-treatment, but no histology was performed to prove this statement. Excitable neural elements may regrow closer to the stimulating electrode, resulting in decrease of threshold [60]. Thus, enhancement of neurite outgrowth and inducing a shift to a more bipolar population of SGN, as achieved in our *in vitro* study with the combination of BDNF and CNTF, could be beneficial for CI recipients. Unfortunately, in these two *in vivo* studies BDNF and CNTF were not tested alone. It would be interesting to determine if the increased sensitivity for electrical stimulation is maybe more induced by increased survival rate (BDNF), neurite regeneration (CNTF) or is only detectable with the additive/synergistic effect of the NTF combination. For example, Nakaizumi et al. [36] studied the effect of gene therapy with BDNF and CNTF in the inner ear, induced by injection of adenoviral vectors into the scala tympani of deafened guinea pigs. The survival of SGN was significantly enhanced after treatment with BDNF and the combination of BDNF and CNTF, but CNTF did not protect the SGN by itself nor did it significantly enhance the protective effect of BDNF. Unfortunately, they did not perform eABRs to control the effect of the combination of both NTFs on the electrical excitability, which was highly improved at the studies of Shinohara et al. and Yamagata et al. Additionally, Nakaizumi et al. did not mention the productivity of the transfected cells, so maybe the CNTF concentration was too low for a protective effect on the SGN. In our study, we could show that a higher concentration of CNTF enhances the survival *in vitro* and both, Shinohara et al. and Yamagata et al., used a high CNTF concentration for their studies.

Nevertheless, none of the *in vivo* studies investigated the effect of the neurite regeneration towards the electrode array that might be an important result of the simultaneous application of BDNF and CNTF, as Shinohara et al. and Yamagata et al. indicate. Recent studies [41,42,60] could show a positive effect on the regrowth and on implant performance.

In conclusion, the combination of high doses of BDNF and CNTF is not only beneficial for an enhanced SGN survival *in vitro* [and *in vivo* [58,59]], but also bears an immense potential for neurite outgrowth and protection or induction of a bipolar morphology of neurons in dissociated spiral ganglion culture *in vitro*. Further experiments should concentrate on *in vivo* experiments in order to demonstrate a putative neurite guidance potential of the herein presented *in vitro* treatment regimen. If so, there is a huge potential to improve especially the nerve electrode interface with benefits like lower thresholds [58,59], increased dynamic range of function and a greater selectivity of excitation in a bioactive CI.
